# Prognostic value of tumor-infiltrating FoxP3^+^ regulatory T cells in cancers: a systematic review and meta-analysis

**DOI:** 10.1038/srep15179

**Published:** 2015-10-14

**Authors:** Bin Shang, Yao Liu, Shu-juan Jiang, Yi Liu

**Affiliations:** 1 Department of thoracic surgery, Provincial Hospital Affiliated to Shandong University, Jinan, Shandong, 250021, China; 2 Department of Respiratory Medicine, Provincial Hospital Affiliated to Shandong University, Jinan, Shandong, 250021, China

## Abstract

The prognostic value of FoxP3^+^ regulatory T cells (Tregs) in cancer remains controversial. We did a meta-analysis to assess the prognostic effect of FoxP3^+^ Treg across different types of cancer and to investigate factors associated with variations in this effect. PubMed, Embase, Cochrane CENTRAL, and Scopus were searched to identify eligible studies. In total, we analyzed 76 articles encompassing 17 types of cancer, and including 15,512 cancer cases. The overall pooled analysis including all types of cancer suggested FoxP3^+^Tregs had a significant negative effect on overall survival (OS) (OR 1.46, P < 0.001), but the prognostic effect varied greatly according to tumor site. High FoxP3^+^ Tregs infiltration was significantly associated with shorter OS in the majority of solid tumors studied, including cervical, renal, melanomas, and breast cancers, *et al*; whereas, FoxP3^+^ Tregs were associated with improved survival in colorectal, head and neck, and oesophageal cancers. The stratified analysis suggested the molecular subtype and tumor stage significantly influenced the prognostic value of FoxP3^+^ Tregs in certain types of cancer. In conclusion, our meta-analysis suggests that the prognostic role of FoxP3^+^ Tregs was highly influenced by tumor site, and was also correlated with the molecular subtype and tumor stage.

The prognostic significance of tumor-infiltrating lymphocytes (TILs) has been a longstanding topic of debate. Many studies across a wide variety of human tumors have shown a significant association between the presence of TILs and patient survival^1^,^2^,^3^. However, it is important to distinguish between different types of T lymphocytes, because they may play different role in the tumor microenvironment. Most TILs have a CD3^+^ phenotype, and CD3^+^ TILs can be further subdivided into cytotoxic (CD8^+^) T cells, memory (CD45RO^+^) T cells, and regulatory (CD4^+^ CD25^+^) T cells^4^,^5^. Cytotoxic (CD8^+^) T cells are thought to have antitumor functions, therefore, a high density of CD8^+^ T cells has been shown to be associated with improved outcome^6^,^7^. The existing evidence suggests that regulatory T cells (Tregs) have the ability to inhibit host-versus tumor immunity in the tumor microenvironment via suppression of antitumor cytotoxic T cells^5^,^8^,^9^.

The transcription factor forkhead box P3 (FoxP3) is a key intracellular molecule for Tregs development and function^10^, which is considered to be the most specific Tregs marker so far. Under normal conditions, FoxP3^+^ Tregs are essential suppressors of antitumor responses and thus maintain immunological tolerance to host tissues^11^. High infiltration of FoxP3^+^ Tregs is expected to be associated with an unfavorable outcome. Thus, FoxP3^+^ Tregs are investigated as a potential prognostic factor and they may also represent a novel therapeutic target. Indeed, this relation has been reported in a wide range of localized or metastatic human carcinomas, including breast^12^, ovarian^13^, hepatocellular^14^, lung^15^, gastric^16^ and cervical cancers^17^. However, recent studies reported increased frequency of FoxP3^+^ Tregs was associated with improved prognosis in some tumors^18^,^19^, such as colorectal cancer. So far the results are stilling conflicting, whether the prognostic effect of FoxP3^+^ Tregs attributable to the biologic properties of specific cancer type, and whether the associations depend on differences in study methodologies was not known.

These studies have been carried out across many types of cancer, with widely differing sample sizes. We were interested in obtaining a reliable conclusion of the prognostic effect of FoxP3^+^ Tregs. Therefore, we conducted a systematic review and meta-analysis, aiming to establish pooled estimates for survival outcomes based on the presence of intratumoral FoxP3^+^ Tregs infiltrating in different types of cancer.

## Methods

We conducted and reported this systematic review and meta-analysis following the PRISMA statement^20^.

### Search strategy and selection criteria

We systematically searched PubMed, Embase, Cochrane CENTRAL, and Scopus (from their commencements to December 2014), with no language restrictions, for studies in humans of the prognostic significance of FoxP3^+^ Tregs in solid tumor. Haematological malignancies were excluded, because these are malignancies of the immune cells themselves. The following keywords were used in searching: (‘regulatory T cells’ or ‘FoxP3’) and (‘prognosis’ or ‘mortality’ or ‘survival’). We scrutinised the reference lists of the identified reports, reviews, meta-analyses, and other relevant publications to find additional pertinent studies. The “related articles” function was used to broaden the search.

### Inclusion and exclusion criteria

Studies that met the following criteria were included in the meta-analysis: studies must have (1) been published as original articles; (2) evaluated human subjects; (3) FoxP3^+^ Tregs in tumor surgical specimens was evaluated using immunohistochemical method; (4) reported association of high and low FoxP3^+^ Tregs infiltration with overall survival (OS), and/or disease-free survival (DFS), or relapse-free survival (RFS); and (5) contained the minimum information necessary to estimate the effects (i.e., hazard ratio) and a corresponding measure of uncertainty (i.e., confidence interval, P-values, standard errors or variance). As an additional criterion, when a single population was reported in multiple reports, only the report with the most complete data was included to avoid duplication.

The eligibility of each study was assessed independently by two investigators (BS and YL). We excluded studies that were not published as full reports, such as conference abstracts and letters to editors, studies that not report sufficient data to estimate survival rates; studies of only peritumoural or peripheral blood analysis.

### Data extraction

Two investigators (BS and YL) independently summarized the studies meeting the inclusion criteria, and performed data extraction using a predefined form, recording: author, journal, year of publication, sample size, tumor type, median follow-up time, impact factor, scoring protocols to identify FoxP3^+^ Tregs, outcome of univariate and/or multivariate analysis (including P-values, hazard ratios, and 95% confidence intervals).

### Measures

The endpoint used in this meta-analysis is OS and DFS (or RFS). The definition of DFS is the period after curative treatment when no disease can be detected. For studies included patients treated with tumor resection, DFS was substituted by RFS. RFS refer to the period after surgical resection when no disease can be detected. For FoxP3^+^ Tregs, study-defined binary variables indicating either the presence (versus absence), positive (versus negative), or high (versus low) expression were used and described as “high” or “low” FoxP3^+^ Tregs infiltrating for this meta-analysis.

### Statistical analyses

Our meta-analysis and statistical analyses were performed with Revman software (version 5.2; Cochrane Collaboration, Oxford, United Kingdom). Odds ratio (OR) and its 95% confidence interval (CI) were used to estimate the association between FoxP3^+^ Tregs and patients’ prognosis. If results of both univariate and multivariate Cox regression analyses were reported, we used estimates from the multivariate Cox regression model for a more direct estimate of the effect of FoxP3^+^ Tregs after controlling for potential confounding variables. If a direct report of survival and recurrence ratios were not available, then the survival data read from Kaplan-Meier curves were read by Engauge Digitizer version 4.1 (http://digitizer.sourceforge.net/) as described previously^21^. This work was performed by two independent reviewers (BS and YL) to reduce inaccuracy in the extracted survival rates.

We assessed heterogeneity between studies with the I^2^ statistic as a measure of the proportion of total variation in estimates that is due to heterogeneity^22^, where I^2^ values of 25%, 50%, and 75% correspond to cut-off points for low, moderate, and high degrees of heterogeneity. Subgroup analyses were carried out to investigate potential sources of between study heterogeneity and to assess whether conclusions were sensitive to restricting studies to subgroups that might have different prognostic effects. Subgroups were defined according to different types of cancer, FoxP3^+^ Tregs scoring strategies (use of tissue microarrays versus whole sections), follow-up duration (a shorter-term versus a longer-term), multivariate corrected or not, molecular subtype and tumor stage for certain types of cancer. Tests for effects subgroup interaction were performed.

## Results

**Figure 1:**
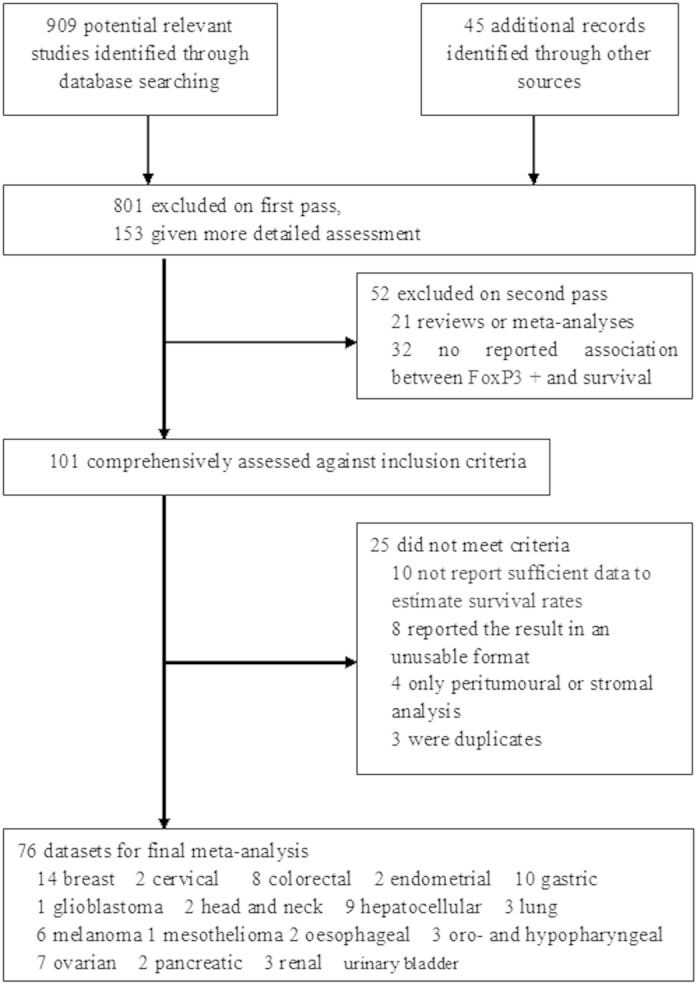
Flow diagram of search strategy and study selection.

Of 4285 citations, we identified 76 articles encompassing 17 different cancer types which met the inclusion criteria, including breast, cervical, colorectal (colon and rectum), endometrial, gastric, glioblastoma, head and neck, hepatocellular, lung, malignant melanoma, oesophageal, oro- and hypopharyngeal, ovarian, pancreas, pleural mesothelioma, renal and urinary bladder cancers (Table 1). Figure 1 showed our search and selection process. Agreement between observers on which studies to include was good (Cohen’s unweighted κ = 0.86). All papers used in our analysis were published in English. Table 1 summarizes some important study characteristics. The analysis included 15512 cancer cases, over more than 108000 personyears of follow-up. The largest number of studies focused on breast cancer (14 studies), and then the gastrointestinal cancers (colorectal cancer, 8 studies; gastric cancer, 10 studies; and hepatocellular carcinoma, 9 studies). The sample size for per cancer site varied from 32 (pleural mesothelioma) to 5183 (breast cancer). The majority of the papers (46/76) were published since 2010. Tissue microarrays were used in 12 of the 76 studies, and other studies used whole-tissue slides to evaluate FoxP3^+^ Tregs. The median number of FoxP3^+^ Tregs was used as the cutoff point in all of the component studies. The mean follow-up per cancer site varied from 1.17 years (head and neck cancer) to 10.55 years (breast cancer), and the mean impact factor ranged from 2.15 (head and neck cancers) to 8.69 (colorectal cancer); 50 studies used multivariate model to assess the prognostic significance of FoxP3^+^ Tregs (Table 1).

### The prognostic effect of FoxP3^+^ Tregs on survival in all types of cancer

OS was reported in 59 studies with a total of 12563 cancer patients. The meta-analysis of all these studies confirmed a significant association between FoxP3^+^ Tregs and survival, high FoxP3^+^ Tregs density was associated with a significant lower OS rate with a pooled OR of 1.46 (95% CI 1.19 to 1.78, P = 0.0002) (Supplementary Figure). A high statistical heterogeneity between studies was noted (I^2^ = 81%, P < 0.001). The data for DFS was reported in 37 studies including 8460 cancer patients. The pooled analyses for DFS were similar, both pointing to a decreased survival associated with high FoxP3^+^ Tregs infiltration (OR 1.23, 95% CI of 1.01 to 1.50, P = 0.003). Statistical heterogeneity was also observed among the studies (I^2^ = 77%, P = 0.0003).

### Subgroup analysis on the prognostic effect of FoxP3^+^ Tregs for different cancer types

**Figure 2:**
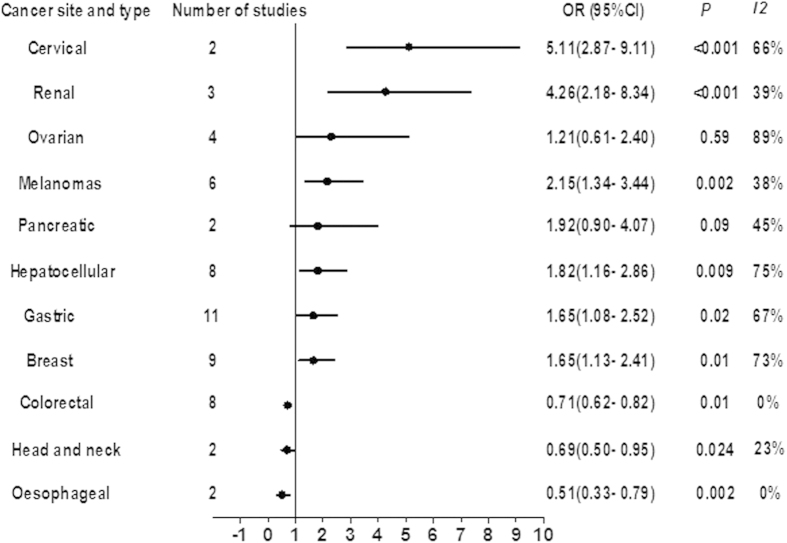
Summary risk estimates of overall survival (OS) by cancer sites.

Figure 2 shows the results of meta-analyses of OS for each tumor site. High FoxP3^+^ Tregs densities were associated with significantly shorter OS in cervical (OR 5.11, 95% CI 2.87 to 9.11, P < 0.001), renal (OR 4.26, 95% CI 2.18 to 8.34, P < 0.001), melanomas (OR 2.15, 95% CI 1.34 to 3.44, P = 0.002), hepatocellular (OR 1.82, 95% CI 1.16 to 2.86, P = 0.009), gastric (OR 1.65, 95% CI 1.08 to 2.52, P = 0.02), and breast cancers (OR 1.65, 95% CI 1.13 to 2.41, P = 0.13). No significant difference was observed in the OS rate for pancreatic (OR 1.92, 95% CI 0.90 to 4.07, P = 0.09), and ovarian (OR 1.21, 95% CI 0.61 to 2.40, P = 0.585) cancer, but there was a trend toward lower survival in pancreatic cancer patients with higher density infiltration of FoxP3^+^ Tregs. Conversely, increased infiltration of FoxP3^+^ Tregs was found to be associated with improved OS in colorectal (OR 0.71, 95% CI 0.62 to 0.82, P = 0.01), head and neck (OR 0.69, 95% CI 0.50 to 0.95, P = 0.024), and oesophageal cancer (OR 0.51, 95% CI 0.33 to 0.79, P = 0.002). Between-study heterogeneity was high for cervical, ovarian, hepatocellular, gastric, and breast cancers (I^2^ ≥ 50%), and moderate or low for the other sites (I^2^ < 50%) (Fig. 2).

In a stratified analysis for breast cancer, the prognostic significance of FoxP3^+^ Tregs varied based on the estrogen receptor (ER) status. FoxP3^+^ Tregs were strongly associated with reduced lower OS rate in ER^+^ breast cancer cases, with a pooled OR of 1.50 (95% CI 1.17 to 1.93), but was associated with a improved survival in ER- cases, with a pooled OR of 0.45 (95% CI 0.31 to 0.65). For ovarian cancer, the prognostic significance of FoxP3^+^ Tregs varied based on disease stage, in high grade or advanced ovarian cancer, high numbers of FoxP3^+^ Tregs infiltrating were found to be associated with improved survival (OR 0.34, 95% CI 0.21 to 0.56, P < 0.001), whereas the pooled analysis of the remaining studies showed a poor prognostic effect of FoxP3^+^ Tregs (OR 2.29, 95% CI 1.07 to 4.90, P = 0.033) (Table 2).

**Figure 3:**
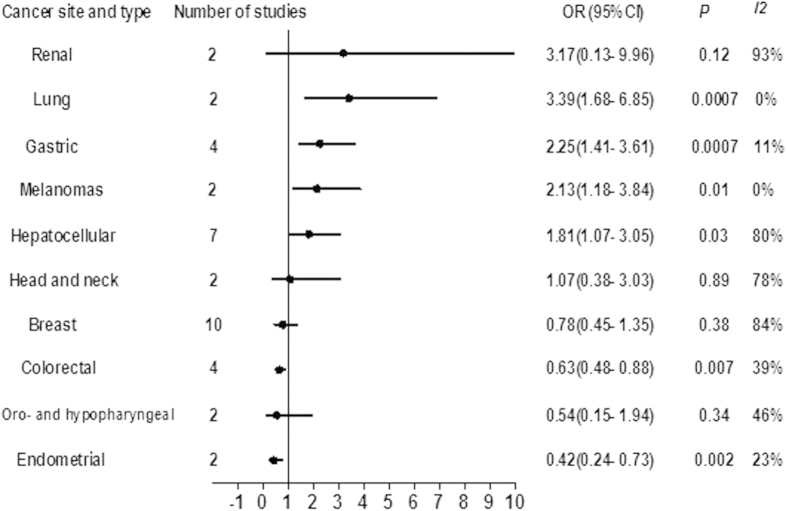
Summary risk estimates of disease-free survival (DFS) by cancer sites.

Figure 3 shows the results of meta-analyses of DFS for each site. High densities of FoxP3^+^ Tregs were significantly associated with lower DFS rate in lung (OR 3.39, 95% CI 1.68 to 6.85, P < 0.001), gastric cancer (OR 2.25, 95% CI 1.41 to 3.61, P < 0.001), melanomas (OR 2.13, 95% CI 1.18 to 3.84, P = 0.01), and hepatocellular carcinoma (OR 1.81, 95% CI 1.07 to 3.05, P = 0.03). FoxP3^+^ Tregs were not significantly associated with DFS in renal (OR 3.17, 95% CI 0.13 to 9.96, P = 0.12), head and neck (OR 1.07, 95% CI 0.38 to 3.03, P = 0.89), breast cancers (OR 0.78, 95% CI 0.45 to 1.35, P = 0.38), and oro- and hypopharyngeal carcinoma (OR 0.54, 95% CI 0.15 to 1.94, P = 0.34). High FoxP3^+^ Tregs infiltration was associated with significantly longer cancer-specific survival in colorectal cancer (OR 0.63, 95% CI 0.48 to 0.88, P = 0.007) and endometrial cancer (OR 0.42, 95% CI 0.24 to 0.73, P = 0.002). Statistical heterogeneity among the studies was high for renal, hepatocellular, head and neck, and breast cancers (I^2^ ≥ 50%), and moderate or low for the other sites (I^2^ < 50%) (Fig. 2).

### Subgroup analysis according to FoxP3^+^ Tregs scoring strategies, follow-up duration

In the stratified analysis, we examined whether the tissue used for FoxP3^+^ Tregs scoring affected estimates of the association between FoxP3^+^ Tregs and survival. Studies using whole-tissue slides showed FoxP3^+^ Tregs were significantly associated with lower OS (OR 1.47, 95% CI 1.22 to 1.77, P < 0.001), whereas the pooled results from studies using tissue microarrays showed FoxP3^+^ Tregs were not significantly associated with OS (OR 0.79, 95% CI 0.62 to 1.01, P = 0.06) (Table 2).

We also examined whether results differed according to duration of follow-up. Studies with a long term follow-up (>5 years) showed a statistically significant pooled result of OS (OR 1.57, 95% CI 1.24 to 1.97, P < 0.001) rate; the pooled results from studies with a short term follow-up showed FoxP3^+^ Tregs were also significantly associated with OS (OR 1.22, 95% CI 1.08 to 1.38, P = 0.01). However, follow-up duration was not reported in all studies (Table 2).

### Risk adjusted analysis

Results of multivariate adjusted analysis for OS were reported in 41 studies. The risk-adjusted analysis confirmed a significant association between FoxP3^+^ Tregs and OS, with a pooled OR of 1.38 (95% CI 1.04 to 1.83, P = 0.025). The results of multivariate adjusted analysis for DFS were only available in 16 studies, the combined risk-adjusted analysis showed FoxP3^+^ Tregs were not significantly associated DFS by multivariate analysis (OR 0.76, 95% CI 0.48 to 1.21, P = 0.25).

### The prognostic effect of the ratio of cytotoxic CD8^+^ T cells to FoxP3^+^ Treg

Relatively few studies incorporated ratio of other T-lymphocyte to FoxP3^+^ Treg. Moreover, the use of different survival outcomes (overall, disease-specific, disease-free, and relapse-free survival) decreased the potential for pooled analysis even further. Therefore, pooled analysis was only possible for the CD8^+^/FoxP3^+^ Treg ratio. The meta-analysis of 8 studies including 1094 patients suggested a high CD8^+^/FoxP3^+^ Treg ratio was significantly associated with improved OS (OR 0.51, 95% CI 0.30 to 0.88, P = 0.02).

## Discussion

While cytotoxic TILs are generally associated with favorable clinical outcome in various tumor settings^1^, studies of the prognostic value of FoxP3^+^ Tregs have lead to highly discrepant results. Our large meta-analysis comprehensively reviewed 76 studies on the prognostic significance of tumor-infiltrating FoxP3^+^ Tregs in 17 types of cancer (15512 cancer cases). The overall pooled analysis of all types of cancer found a negative prognostic effect associated with FoxP3^+^ Tregs preponderance. However, the prognostic value of FoxP3^+^ Tregs varied significantly according to carcinoma sites. High FoxP3^+^ Tregs infiltration was significantly associated with poor prognosis in the majority of solid tumors studied; no prognostic effect of FoxP3^+^ Tregs were observed in ovarian, pancreatic cancers and *et al.*; whereas, in colorectal, head and neck, and oesophageal cancers, tumor infiltrating FoxP3^+^ Tregs were associated with favorable prognosis. Moreover, the stratified analysis suggested the molecular subtype or tumor stage influenced the prognostic value of FoxP3^+^ Tregs in certain types of cancer, but the prognostic variability may not be attributable to different scoring strategies, follow-up duration, multivariate corrected or not. Therefore, FoxP3^+^ Treg is inadequate as a single prognostic marker.

The finding that a high density of infiltrating FoxP3^+^ Tregs was associated with unfavorable outcome in a wide range of tumors supported the theory that the tumor-infiltrating FoxP3^+^ Tregs could be an escape mechanism of human cancers to the immune response. The association was particularly strong for cervical cancer, and then were renal, lung, melanomas, hepatocellular, and gastric cancers. All the studies of cervical and renal cancer unanimously concluded that FoxP3^+^ Tregs were associated with a poor prognosis^17^,^23^,^24^. Most of the studies looking at melanomas also reported poor prognostic effect^25^,^26^, with the remaining studies showing a neutral prognostic claims^27^,^28^. In renal cancer and melanoma, FoxP3^+^ Tregs infiltration was found to be significantly associated with high cyclooxygenase-2 (COX-2) expression^24^,^26^. It is conceivable that the variability of FoxP3^+^ Tregs prognostication could attribute to the inherent molecular heterogeneity. In support of this idea, our pooled analysis showed the prognostic value of FoxP3^+^ Tregs were stronger in COX-2^+^ cases compared with COX-2^–^ cases. COX-2 has been demonstrated to lead to tumor progression through the promotion of tumor cell survival, invasiveness, and angiogenesis in a variety of tumors^26^. The correlation between high intratumoral COX-2 expression and gathering of FoxP3^+^ Tregs should be more clearly characterized in future studies.

The prognostic value of FoxP3^+^ Tregs in breast cancer is most frequently studied. However, studies of breast cancer have lead to highly discrepant findings^13^,^29^,^30^,^31^. Our pooled analysis of the 13 studies with a total of 5,167 patients suggested high FoxP3^+^ Tregs infiltration was significantly associated with decreased OS in breast cancer. The prognostic claims in the 13 studies ranged from poor (n = 4), to neutral (n = 6), to good (n = 3). The inconsistent results maybe somewhat related to the different subtypes of breast cancer. In the stratified analysis according to ER status, we found high numbers of FoxP3^+^ Tregs was associated with a favorable outcome in ER- breast cancer, but was associated with poor prognosis in ER^+^ breast cancer. Because most of the studies in breast cancer involved mixed cohorts that were largely comprised of ER^+^ cases, the association between FoxP3^+^ Tregs and good prognosis in ER– cases may have been obscured by a negative prognostic relationship among ER^+^ tumors.

For gastrointestinal cancers including hepatocellular carcinoma, colorectal cancer and gastric cancer, the prognostic value of FoxP3^+^ Tregs was completely different. Most of the studies looking at hepatocellular cancer reported poor prognostic effect of FoxP3^+^ Tregs, and the remaining studies reported neutral prognostic claims. Our pooled results showed high density of FoxP3^+^ Tregs infiltrating was associated with poor survival and high recurrences in hepatocellular cancer. The 11 studies of gastric cancer showed a split among poor (n = 3), neutral (n = 6), and good (n = 2) prognostic claims. Base on those studies, our meta-analysis suggested a high density of FoxP3^+^ Tregs was associated with poor survival and high recurrences for gastric cancer. Conversely, most of the studies investigating colorectal cancer concluded FoxP3^+^ Tregs correlated with a good prognosis^32^,^33^. Our pooled analysis of 8 studies involving 3972 patients established that high number of intratumoral FoxP3^+^ Tregs was associated with longer OS and DFS in colorectal cancer.

This correlation between FoxP3^+^ Tregs and favorable clinical outcome has also been observed in oesophageal and head and neck cancers in our meta-analyses. In patients with follicular lymphoma and Hodgkin’s lymphoma, it is now well established that high number of intratumoral FoxP3^+^ Tregs are associated with longer DFS and OS, even in multivariate analyses^34^,^35^. Hematologic malignancies and solid tumors (head and neck cancer and colon cancer), in which presence of FoxP3^+^ Tregs correlate with good clinical outcome, are tumors heavily infiltrated by inflammatory immune cells, such as macrophages and neutrophils, which produce growth factors or inflammatory cytokines favoring tumor progression^36^,^37^. FoxP3^+^ Tregs have been shown to suppress inflammation triggered by innate immune cells in mice^38^. In human cancers, FoxP3^+^ Tregs were shown to kill macrophages and monocytes and suppress their protumor effect^39^. As a result, the positive impact of FoxP3^+^ Tregs may be partially attribute to down regulate an unresolved inflammatory response which could promote tumor progression. In addition, it has been demonstrated that adoptive immunotherapy with CD4^+^CD25^+^Tregs could decrease tumor multiplicity through inducing apoptosis of intestinal tumors^38^. However, the mechanisms leading to the observed correlation between FoxP3^+^ Tregs infiltration and favorable prognosis and its clinical impact remain unclear and warrant further investigation.

In ovarian cancer, the prognostic effect of FoxP3^+^ Tregs has been inconsistently reported, ranged from poor (n = 3), to neutral (n = 1), to good (n = 2)^2^,^3^,^12^,^40^,^41^. Our pooled analysis suggested FoxP3^+^ Tregs were not a significant prognostic indicator. Barnett *et al.* observed there may be an association between increased FoxP3^+^ Tregs infiltration and advanced stage in ovarian cancer^41^. Our stratified analyses demonstrated that the prognostic value of FoxP3^+^ Tregs in ovarian cancer varied depending on tumor stages. In high grade or advanced ovarian cancer, high numbers of FoxP3^+^ Tregs infiltrating were found to be associated with improved survival, whereas the pooled analyses of the remaining studies showed FoxP3^+^ Tregs were associated with poor prognosis.

Given that the prognostic impact of Tregs appears to differ based on tumor sites, molecular subtype, and tumor stage, it should be cautious about the strategies aimed at depleting or inhibiting these cells to enhance tumor immunity. Treg depletion has been tested as a therapeutic approach in animal models and clinical trials, for instance by treatment with low doses of cyclophosphamide to destroy Tregs, restored efficacy of immunotherapy^42^. A clinical study targeted CD25 for depletion of Tregs in advanced nonsmall cell lung cancer (NSCLC) showed limited treatment effect^43^. In contrast, slective depletion of Foxp3^+^ regulatory T cells improves effective therapeutic vaccination against established melanoma^44^. The inconsistent findings as well as our results suggest that this strategy may be beneficial for some tumor sites but not for others. Until we have a better understanding of the functional properties of FoxP3^+^ Tregs, it seems premature to proceed with strategies aimed at depleting these cells in patients.

Several studies made prognostic claims on the basis of the ratio of FoxP3^+^ Tregs to other lymphocyte subsets, and the CD8^+^/FoxP3^+^ T cell ratio was commonly used. Based on these studies, our meta-analysis indicated that a high CD8^+^/FoxP3^+^ T cell ratio was independently associated with improved survival. Previous studies showed FoxP3^+^ Tregs were strongly associated with effector T cells and maybe an indicator of a strong CD8^+^ T cell response, which might outweigh any immunosuppressive effects of FoxP3^+^ Tregs^3^,^15^,^45^,^46^. In this point of view, infiltration of tumor epithelium, or any other inflammatory site, by lymphocytes is naturally accompanied by FoxP3^+^ Tregs and the effectiveness of immune responses depends on the proportion of the different lymphocyte subtypes present instead of on the presence of a particular subtype. Further studies of the ratio of FoxP3^+^ Tregs to different tumor infiltrating lymphocytes (CD8, CD3) could add insight into the immunologic microenvironment associated with immune evasion.

Although we believe that the current meta-analysis provided useful information, some potential limitations should be addressed. Firstly, heterogeneity in our study is substantial and may be attributed to differences in types of cancer, cell scoring strategies, study era, treatment strategies, and so on, which restricted us obtaining more comprehensive results. Moreover, because the prognostic role of FoxP3^+^ Treg seems to be substantially different according to tumor site, the overall pooled analysis of all types of cancer maybe highly dependent on the relative proportion of each specific type of cancer. This provided associative, not evidence and mandates caution when interpreting this result.

In conclusion, we have demonstrated the prognostic value of FoxP3^+^ Tregs may not equivalent in different tumors. Thus, the original view that FoxP3^+^ Tregs invariably suppress tumor immunity is oversimplified. The discrepant prognostic effect of FoxP3^+^ Tregs could arise from different biologic properties of specific tumor types, and the positive impact may be related to their anti-inflammatory effects in several tumors. Moreover, in many cancers, the prognostic effect of FoxP3^+^ Treg is highly correlated with tumor stage or molecular subtype. Further improved understanding of FoxP3^+^ Treg subsets in different human cancers will likely enable the development of more precise and effective immunotherapies.

## Additional Information

**How to cite this article**: Shang, B. *et al.* Prognostic value of tumor-infiltrating FoxP3^+^ regulatory T cells in cancers: a systematic review and meta-analysis. *Sci. Rep.* **5**, 15179; doi: 10.1038/srep15179 (2015).

## Supplementary Material

Supplementary Information

## Figures and Tables

**Table 1:** Baseline characteristics for studies included in meta-analysis.

Types of cancer	Number of Studies	Year of publication		Sample size	Cutoff points		Method for detecting Treg		Multivariate Adjusted	Mean Impact factor	Mean duration of follow-up (years)
		Before 2010	2010–2014		Absence vs presence	High vs low	tissue microarrays	whole sections			
Breast cancer	14	3	11	5183	3	11	3	11	10	6.78	10.55 years
Cervical cancer	2	1	1	155	0	2	0	2	1	5.63	5 years
Colorectal cancer	8	2	6	3972	0	8	4	4	6	8.69	6.54 years
Endometrial cancer	2	2	0	447	0	2	1	1	1	3.93	4.64 years
Gastric cancer	10	2	8	1149	0	10	1	9	6	4.23	4.09 years
Glioblastoma	1	0	1	62	0	1	0	1	1		1.46 years
Head and neck cancers	2	1	1	167	0	2	0	2	2	2.15	1.17 years
Hepatocellular carcinoma	9	5	5	1419	0	9	0	9	6	5.05	4.47 years
Non-small cell lung cancer	3	1	2	251	0	3	0	3	1	3.93	3.17 years
Melanomas	6	2	4	528	0	6	0	6	2	3.32	6.39 years
Oesophageal carcinoma	2	1	1	252	0	2	0	2	2	3.85	5 years
Oro- and hypopharyngeal carcinoma	3	2	1	235	0	3	2	1	2	2.51	6.37 years
Ovarian Cancer	7	5	2	869	0	7	0	7	3	3.69	4.53 years
Pancreatic carcinoma	2	1	1	260	0	2	0	2	2	4.09	1.75 years
Pleural mesothelioma	1	1	0	32	0	1	1	0	1	3.53	5 years
Renal cancer	3	2	1	494	0	3	0	3	3	4.61	4.25 years
Urinary bladder cancer	1	0	1	37	0	1	0	1	1	3.05	3 years

**Table 2:** Subgroup analysis of the prognostic significance of FoxP3^+^ Treg

	No. of studies	No. of patients	OR (95% CI)	Overall effect P-value	Subgroup difference P-value	I^2^
Breast					<0.001	
All studies	9	3521	1.65 (1.13 to 2.41)	0.01		73%
Studies of ER^+^ cases	2	1682	1.50 (1.17 to 1.93)	0.03		30%
Studies of ER- cases	2	278	0.45 (0.31 to 0.65)	<0.001		0%
Ovarian cancer					<0.001	
All studies	6	794	1.21 (0.61 to 2.40)	0.59		89%
Studies of advanced stage cases	2	306	0.34 (0.21 to 0.56)	<0.001		0%
Other studies	4	488	2.29 (1.07 to 4.90)	0.033		89%
Cyclooxygenase-2 (COX-2) expression					<0.001	
COX-2^+^ cases	3	287	3.62 (2.37 to 5.51)	<0.001		0%
Other studies	58	12276	1.33 (1.13 to 1.57)	0.06		89%
Tissue used					<0.001	
Whole-tissue slides	55	7712	1.47 (1.22 to 1.77)	<0.001		82%
Tissue microarrays	6	4854	0.79 (0.62 to 1. 01)	0.06		66%
Follow-up					0.13	
Short term (≤5 years)	17	3559	1.22 (1.08 to 1.38)	<0.001		67%
Long term (>5 years)	38	8147	1.57 (1.24 to 1.97)	0.001		43%
Multivariate correction					0.31	
Yes	41	9881	1.38 (1.04 to 1.83)	0.025		74%
No	20	2395	1.52 (1.02 to 2.26)	0.04		39%
T-lymphocyte ratios						
CD8^+^/FoxP3^+^ T cell ratio	8	1094	0.51 (0.30 to 0.88)	0.02		37%
