# Supplementary material for: Prognostic value of tumor-infiltrating FoxP3+ regulatory T cells in cancers: a systematic review and meta-analysis
Source: Sci Rep. 2015 Oct 14;5:15179. doi: 10.1038/srep15179 (PMC4604472; doi:10.1038/srep15179)
Supplement: Supplementary Information [file srep15179-s1.doc]

**Prognostic value of tumor-infiltrating FoxP3^+^ regulatory T cells in cancers: a systematic review and meta-analysis**

*Bin Shang^1^, Shu-juan Jiang^2^, Yi Liu^2^, Yao Liu^2^**

Institution of authors: 1. Department of thoracic surgery, Provincial Hospital Affiliated to Shandong University, Jinan, Shandong, 250021,China

2. Department of Respiratory Medicine, Provincial Hospital Affiliated to Shandong University, Jinan, Shandong, 250021,China

*Corresponding author: Yao Liu E-Mail: doctorliuyao@126.com

**Supplementary Figure:** Meta-analysis of the impact of FoxP3+ Tregs on overall survival in all types of cancer
